# Management of Recurrent Venous Thromboembolism on Anticoagulation

**DOI:** 10.3390/jcm15093415

**Published:** 2026-04-29

**Authors:** Jennifer Eatrides, Avani Singh, Ankita Patel, Michael Jaglal, Geetha Rajasekaran Rathnakumar, Enas Abdallah, Damian A. Laber

**Affiliations:** 1 Department of Ambulatory Hematology and Oncology, H. Lee Moffitt Cancer Center, Tampa, FL 33607, USAdamian.laber@moffitt.org (D.A.L.); 2 Department of Oncologic Sciences, University of South Florida, Tampa, FL 33612, USA; 3 Department of Malignant Hematology, H. Lee Moffitt Cancer Center, Tampa, FL 33612, USA

**Keywords:** DOAC failure, recurrent VTE, thromboembolism on anticoagulation

## Abstract

Direct oral anticoagulants (DOACs) are the standard first treatment for patients with venous thromboembolism. Unfortunately, some patients develop recurrent thromboembolism despite adherence to anticoagulation. This remains a significant clinical challenge with no randomized data to guide therapy. This review summarizes the available evidence for the management of recurrent venous thromboembolism (VTE) and DOAC failure, and we propose our group consensus and management algorithm.

## 1. Introduction

Venous thromboembolic disease (VTE) is the formation of blood clots in both deep vein thrombosis (DVT) and pulmonary embolism (PE). Superficial vein thrombosis is not considered part of VTE. According to the American Society of Hematology (ASH) guidelines, the first-line treatment for acute VTE is a direct oral anticoagulant (DOAC), unless contraindicated by conditions such as severe renal insufficiency, moderate to severe liver disease, or antiphospholipid antibody syndrome. Other anticoagulants commonly used for outpatients are low-molecular-weight heparins (LMWH), fondaparinux and vitamin K antagonists (VKA). In our clinical practice, we generally initiate a DOAC unless a patient presents with a contraindication.

Recurrent thromboembolic events while on anticoagulation remain a significant clinical challenge. Prospective randomized trials addressing this issue are lacking, leaving current management strategies reliant on observational data, anecdotal experience, and individual physician preference. Comprehensive consensus guidelines are lacking and only recently did AHA/ACC/ACCP/ACEP/CHEST/SCAI/SHM/SIR/SVM/SVN include management of recurrent PE on anticoagulation in their 2026 guidelines for evaluation and management of PE; however, this does not include other VTE sites [1].

Incidence of PE in the U.S. is about 370,000 per year (60–120 per 100,000 people), leading to 60,000 to 100,000 deaths per year [2]. In patients who are compliant with anticoagulation use, the reported incidence of recurrent VTE in large clinical trials for DOACs is about 2–3% [1,3,4,5,6]. Suggested mechanisms of failure include drug interactions, decreased absorption (specifically dabigatran), increased renal clearance (rivaroxaban and edoxaban), and peaks/troughs with once daily dosing (rivaroxaban) [7]. Other reasons for potential breakthroughs include underlying disease such as malignancy, autoimmune disease and unrecognized acquired or hereditary hypercoagulable states.

The aim of this review is to assess the current data and clinical strategies related to managing recurrent VTE on anticoagulation. We propose a framework that may serve as a basis for best practices and future research. Given that DOACs are the current standard of care, our focus is on treatment failure in patients receiving these agents.

## 2. Materials and Methods

We completed a search in PubMed, a search developed by the National Center for Biotechnology Information (NCBI) at the U.S. National Library of Medicine (NLM), that covers MEDLINE journals and provides links to full-text articles via the PubMed Central (PMC) engine and database providing access to citations and abstracts of biomedical and life sciences literature. Since apixaban, rivaroxaban and dabigatran were approved by the FDA for the treatment of VTE in 2014, we searched for studies published from 2015 to 2025 with search terms including DOAC failure, recurrent venous thromboembolism, and thrombosis despite anticoagulation. The authors performed individual searches, and the results were presented to the whole group for selection and inclusion in this manuscript. Manuscripts presenting data from individual patients were selected for inclusion into this manuscript irrespective of the type of study being retrospective or prospective, single or multiple institution, randomized or not. We acknowledge the limited published data and the descriptive nature of our project. Where data were very limited, we included our opinion that included a combined experience of approximately 100 author/years in consulting and managing these patients. Our goal was to understand the problem and promote logical strategies to apply to the daily clinical management of these patients and hopefully collect the results of these strategies to stimulate future research to improve outcomes.

## 3. Risk Factors for VTE

The risk of venous thromboembolism (VTE) in the general population is around 1–2 cases per 1000 person years [8]. Provoking factors include a major surgery or trauma (both strong transient risk factors), immobilization, malignancy, hospitalization, pregnancy, hormone replacement and acute medical illness; however, 40% of patients have no identifiable provoking factor [8]. Excluding active or progressive cancer, the risk of recurrent thrombosis despite anticoagulation is highest in those patients with unprovoked VTE, with an incidence of 1.41 per 100 person years and 5-year incidence of 7.1% [9]. Malignancy is not only a risk factor for VTE but also for breakthrough thrombosis with a 6-month incidence of 5–8% [10]. All risk factors that predispose to VTE should be identified and categorized as reversible or non-reversible. The reversible risk factors should be subclassified as resolved, not yet resolved, or unable to be resolved/permanent.

## 4. Mechanism of Anticoagulation in VTE

Anticoagulants function by inhibiting various components of the coagulation cascade. In the context of VTE, anticoagulants reduce fibrin formation by targeting specific clotting factors and prevent clot propagation or embolization. This allows the body time to resolve the clot by multiple physiologic processes including enzymatic degradation starting with tissue plasminogen activator (tPA) released from endothelial cells, plasmin activation, and fibrinolysis. Macrophages, neutrophils and endothelial cells release enzymes and help to degrade the clot, a process that takes several months. Multiple classes of anticoagulants are used in VTE management, each with distinct mechanisms of action. The duration of anticoagulation depends on the underlying risk for recurrence, with at least 3 months recommended for surgical or other strong provoking transient risk factors and indefinite prolonged (at least 6 months or more) for recurrent or unprovoked events. Reduced-dose DOACs (apixaban 2.5 mg twice daily or rivaroxaban 10 mg daily) are preferred for extended therapy after 6 months of anticoagulation in patients with persistent risk factors [11,12,13,14].

## 5. Definition of Recurrent VTE on Anticoagulation

We define recurrent VTE on anticoagulation as new thromboses that occur while patients are taking an anticoagulant medication at the recommended treatment dose for the DVT or PE. Indicators of recurrent VTE include new thrombosis in a different location or propagation of an existing thrombus.

## 6. How to Diagnose Recurrent VTE on Anticoagulation

In clinical practice, this can often be difficult to assess as not all areas are imaged on initial assessment, and often imaging is completed at different medical centers; thus, it is difficult to accurately compare studies. This is typical for ultrasound evaluations since the probe is placed in different locations during the procedure by an ultrasound technologist utilizing a local algorithm. Symptoms alone should not be used to diagnose recurrent VTE as there are no validated algorithms to identify thrombus age by clinical factors alone. Increasing or new onset swelling of a lower extremity with a prior DVT can be due to post-thrombotic syndrome. While recurrent VTE/PE should be considered in patients with dyspnea or chest pain, further workup is always necessary for these symptoms with a broad differential diagnosis. The diagnosis of recurrence should be confirmed with any of the objective radiologic tests available at the institution with special focus on the image characteristics and the chronicity of the thromboses. Venous duplex ultrasound combines gray-scale imaging with compression with color-flow doppler and waveform analysis [15]. By ultrasound criteria (see Table 1), an acute DVT must be non-compressible with the following secondary criteria: echogenic thrombus in the vein, venous distension, absence of color flow within the vein, loss of flow phasicity, and loss of response to Valsalva or augmentation [15]. Partial venous occlusions and the presence of collateral circulation are mostly due to chronic changes [16]. Body habitus, edema and recent orthopedic surgery can be limiting factors. Computed tomography (CT) angiography (CTA) is the modality of choice to diagnose pulmonary embolism and can be combined with CT venogram to detect DVTs; however, this is not typically the practice at many institutions. Fibrotic bands and venous obliteration are more indicative of a chronic thrombus on CT venogram [16]. Elastography can estimate stiffness of tissue and thus thrombus age, but further research is needed, and it is not currently the standard of care [16]. The American Society of Hematology (ASH) also addresses the workup of recurrent VTE, where the suggested algorithm to identify recurrent VTE starts with assessing clinical likelihood of recurrence, d-dimer and whole-leg compression ultrasound, with full compressibility ruling out recurrent DVT. In cases of non-diagnostic imaging, in an “unlikely” patient, a negative d-dimer can be used to rule out DVT or guide serial imaging in a more “likely” patient [17].

For diagnosis of a new or recurrent pulmonary embolism, we require the blood clot to be in a new location. If images are unclear, we repeat the CTA within a few days before making the final diagnosis of recurrent versus chronic thrombus.

## 7. Causes That Should Not Be Considered Anticoagulation Failure

- Patient nonadherence to the recommendations

The cause of patient nonadherence or poor compliance can be multifactorial and should be clearly documented and managed appropriately. Financial concerns, healthcare literacy, and other complex social determinants of health often contribute to cases of nonadherence.

- Subtherapeutic anticoagulation

In patients receiving warfarin or unfractionated heparin, subtherapeutic anticoagulation is easy to diagnose and treat by utilizing accepted laboratory monitoring. The clinical utility of laboratory monitoring for other anticoagulants has not been proven. In theory, subtherapeutic anticoagulation can occur due to multiple causes including nonadherence, impaired drug absorption or increased metabolism. There are no validated criteria to diagnose these. The specific cause must be identified and managed. Marked obesity and pregnancy have been categorized as risk factors and as a possible cause of subtherapeutic anticoagulation. Laboratory measurement of anti-factor X activity (anti-Xa) or dilute thrombin time (dTT) can be helpful.

- Anatomical causes

Local trauma, hematoma, tumor, local infections, Paget Schroetter syndrome and May–Thurner syndrome can cause obstruction to the blood flow with subsequent thrombus formation.

- Temporary discontinuation of anticoagulation

This can occur in patients undergoing a surgical procedure or after a bleeding complication.

- Other causes that might mimic a clot on imaging studies

Tumor thrombus, fat emboli, carcinomatosis and some infections may look similar to blood clots radiographically and should not be confused, as anticoagulation does not typically have a role in these cases.

- Worsening of the precipitating condition or new risk factor

Progressive malignancy, acute exacerbation of an autoimmune disease, new pregnancy, introduction of pro-thrombotic medication, trauma, or surgery can provoke a new VTE and should be managed accordingly.

- Stable chronic thrombosis

Persistent but stable chronic thromboses should not be considered failure of anticoagulation, thus the importance of comparison to prior imaging to best determine treatment failure. Symptoms alone in this setting may be attributed to post-thrombotic syndrome and should be considered in the absence of imaging confirming acute VTE. D-dimer may also be considered in this setting.

## 8. Laboratory Testing

- For the activity of DOACs

Potential and reasonable motivations for the laboratory testing of DOACs’ activity include breakthrough thrombosis, assessing compliance, workup of suspected impaired drug absorption or increased metabolism, testing prior to surgery, and in the setting of trauma or bleeding [18]. For apixaban, edoxaban, rivaroxaban, an anti-Xa level is available, and for dabigatran dilute thrombin time (dTT) can be checked; however, its role in guiding routine care is uncertain, and studies evaluating trough DOAC levels show significant interpatient variability [19]. Studies in patients with high-risk atrial fibrillation have shown increased thrombotic risk in patients with low DOAC trough levels; however, data are limited [20]. It is important to note that calibrated drug assays are dependent on the specific assay, DOAC drug and dose, and vary between centers. A single-center study reported DOAC monitoring (50% of patients were on DOAC for VTE) with level checked for concern for recurrent VTE in 8% of patients, urgent procedure in 32%, renal failure in 14%, bleeding event in 9% and extremes of body weight in 18%. Clinical decisions were impacted by DOAC monitoring in approximately one-third of the patients and were found to be more helpful to predict bleeding risk [21].

Our center has DOAC-calibrated assays available, and our practice has been to perform this test in cases where poor compliance or decreased absorption are suspected, understanding that there is insufficient evidence to make treatment decisions based on the results. We find the laboratory test results useful when deciding to switch agents in case of decreased absorption and to focus on strategies to increase treatment adherence in cases of poor compliance.

- To clarify equivocal imaging

D-dimer testing can also be incorporated with clinical assessment to help guide the next steps in patients with concern for recurrent VTE, where imaging is equivocal or non-diagnostic. A negative d-dimer in patients who are otherwise “low risk” for VTE can have excellent negative predictive value and can be helpful in ruling out recurrent VTE or treatment failure [17]. This algorithm is suggested for evaluation of recurrent VTE but could also be applied to evaluation of recurrent VTE on anticoagulation as d-dimer is expected to normalize after several months of anticoagulation and acute thrombosis. ISTH also offers similar guidance regarding d-dimer and recommends that d-dimer can be used in cases of equivocal compression ultrasound for evaluation of recurrent DVT [22].

## 9. Literature Review for Management of Recurrent VTE on Anticoagulation

There are no large randomized controlled trials addressing this problem, with the medical literature limited to case reports and retrospective data with limited consensus guidelines specifically in this setting. ASH provides several “How I treat” articles that address the topic of VTE on anticoagulation as well as recurrent VTE in cancer patients. The suggested algorithm for patients who have thrombosis on a DOAC is to change to VKA or LMWH; patients who fail LMWH can escalate to high-dose LMWH. However, this does not address duration or the next steps for patients who decline indefinite parenteral anticoagulation. A similar algorithm is suggested for breakthrough thrombosis in cancer patients, where weight-based LMWH or loading dose of DOAC are both considered options, with reassessment after 1 month [10]. Common practice includes changing therapy or escalating the dose of anticoagulation. This is also supported by recent guidelines that suggest the following related to dose escalation or change in therapy: (1) for patients adherent to therapeutic anticoagulation with recurrent PE, changing to an alternative drug class is reasonable; (2) increasing to full-dose DOAC in the same class is reasonable for patients with recurrence on reduced-dose DOAC; and (3) dose escalation of LMWH by 20–25% is reasonable in cancer patients with recurrent PE despite therapeutic anticoagulation with LMWH [1]. A review of open studies on clinicaltrials.gov shows no active or pending trials evaluating either of these options in the general population. There is a study currently recruiting patients with a concurrent cancer diagnosis and recurrent VTE where LMWH dose escalation is being evaluated (NCT05229471).

In a single-center study that included 54 patients with treatment failure, 84% changed initially to LMWH and were then changed back to oral anticoagulation (19% to VKA, 81% to DOAC) after a median of 52 days, followed by an uncomplicated course. Changing anticoagulation was not reported to prevent post-thrombotic syndrome in 42% of patients with lower-extremity VTE [23]. A recent meta-analysis evaluated 51 manuscripts of patients exhibiting DOAC failure to characterize manifestations of failure and new treatment after failure. Of the 79 patients with treatment failure, the most common failure was in patients with APLS (44.3%). Most patients changed to VKA (55.7%), 10% changed to enoxaparin, and 7.6% changed to DOAC of a different mechanism. Of those changed to a different DOAC, all had resolution of thrombus or no further thromboembolic events, although it is unclear what duration of medical follow-up was achieved [7]. Comparison of the efficacy of dabigatran, rivaroxaban, and apixaban for atrial fibrillation has shown no statistically significant difference in stroke or thromboembolism rate between agents [24]. Although there is no evidence to support a “superior” DOAC, due to patient factors as shown above, the common practice of changing to another DOAC is questionable due to a lack of evidence. When comparing second-line anticoagulants (warfarin, dabigatran, enoxaparin) after failure of apixaban or rivaroxaban, a recent retrospective study found no difference in recurrent thrombosis or bleeding among these second-line options [25].

- Dose-escalation of anticoagulation

As there are no current trials evaluating dose escalation of DOACs, we reflect on the initial trials of DOACs for dose determination. The BISTRO-I and BISTRO-II trials evaluated dabigatran in patients undergoing orthopedic surgery (hip or knee replacement) for prevention of VTE and showed fewer VTE events with higher dosing (OR 0.65 for 150 mg twice a day; OR 0.61 for 300 mg daily; OR 0.47 for 225 mg twice a day) [26,27]. The RENOVATE and REMODEL trials also showed similar results with reduced VTE and mortality with dabigatran 220 mg compared to 150 mg twice a day [28,29]. The RELY trial compared dabigatran to warfarin in patients with atrial fibrillation with fewer thromboembolic events with a higher dose of dabigatran (1.53% per year with 110 mg vs. 1.11% per year with 150 mg twice a day) [30]. However, trials evaluating dose escalation of rivaroxaban for VTE prophylaxis did not show a dose-dependent reduction in VTE [31]. While these trials suggest a potential benefit of dose escalation of dabigatran in a prophylactic setting, they did not evaluate the efficacy of dose escalation in the setting of treatment failure.

Dose escalation of low-molecular-weight heparin to manage recurrent venous thromboembolic events despite systemic anticoagulation was reported in a retrospective cohort study of cancer patients. Forty-seven patients received a 120% dose increase in LMWH. At 3 months, 8.6% had a second recurrent VTE and 4.3% had bleeding complications. The median time between the index recurrent VTE to death was 11.4 months. No long-term data were presented since cancer patients with recurrent VTE have a short median survival [32].

VKA dose escalation was studied in a randomized, double-blind trial in patients with antiphospholipid antibodies and a history of thrombosis. Subjects received warfarin to achieve an INR of 2.0 to 3.0 (standard intensity) or 3.1 to 4.0 (high intensity). Recurrent thrombosis occurred in 10.7% of the individuals assigned to receive high-intensity warfarin and in 3.4% assigned to the standard INR of 2–3. Major bleeding was similar in both arms [33].

- Combined anticoagulation and antiplatelet agents

Another potential strategy in the setting of DOAC failure is the addition of an antiplatelet medication. Several studies have looked at combined platelet and thrombin inhibition, specifically in the setting of cardiovascular disease and peripheral artery disease. Dual pathway inhibition has been shown to have a favorable risk profile and led to FDA approval of the combination of rivaroxaban 2.5 mg BID + aspirin following lower extremity revascularization [34,35]. A recent multicenter randomized clinical trial evaluated patients who had VTE recurrence despite rivaroxaban. Patients were treated with rivaroxaban (20 mg once daily) + aspirin (300 mg once daily) or a dose-adjusted vitamin K antagonist. The study found no statistically significant difference in the groups, but at 90 days follow-up, numerically fewer recurrent thromboembolic events and bleeding events occurred in the rivaroxaban + aspirin group [36]. A meta-analysis also evaluated the combination of anti-platelet therapy with any oral anticoagulant for recurrent VTE and VTE-related death, with a primary safety outcome of major bleeding. Six RCTs were included, none of which were designed specifically to evaluate the role of concomitant antiplatelet therapy and oral anticoagulation but included a subset of patients who were on aspirin (all but one included study excluded patients on >100 mg/day aspirin or >75 mg/day clopidogrel) at the time of randomization. Thirteen percent of patients received antiplatelet therapy, of which 12.6% also took DOACs. Concomitant antiplatelet therapy was not found to reduce the risk of recurrent VTE and was associated with a higher risk of bleeding (major bleed 2.1% vs. 1.1% and non-major bleed 9.4% vs. 6.3% for combination vs. DOACs alone, respectively) [37].

- Other combinations of anticoagulation agents

A small case series study (of eight patients) suggested that combining two DOACs may be considered in refractory cases. However, this approach requires careful consideration due to the potential for increased bleeding risk [38].

- Inferior Vena Cava (IVC) filter

We recommend against the placement of an inferior vena cava (IVC) filter for the long-term management of patients with any VTE since it has shown to increase the risks of recurrent DVT in the absence of anticoagulation [39]. We recommend a temporary placement of an inferior vena cava (IVC) filter with removal within 2 months for patients with a 2–4-week contraindication for therapeutic anticoagulation. When therapeutic anticoagulation is absolutely contraindicated for the rest of the patient’s life, we do not recommend an IVC filter.

## 10. Our Approach to Management of Recurrent VTE on Anticoagulation or DOAC Failure

**Figure 1:**
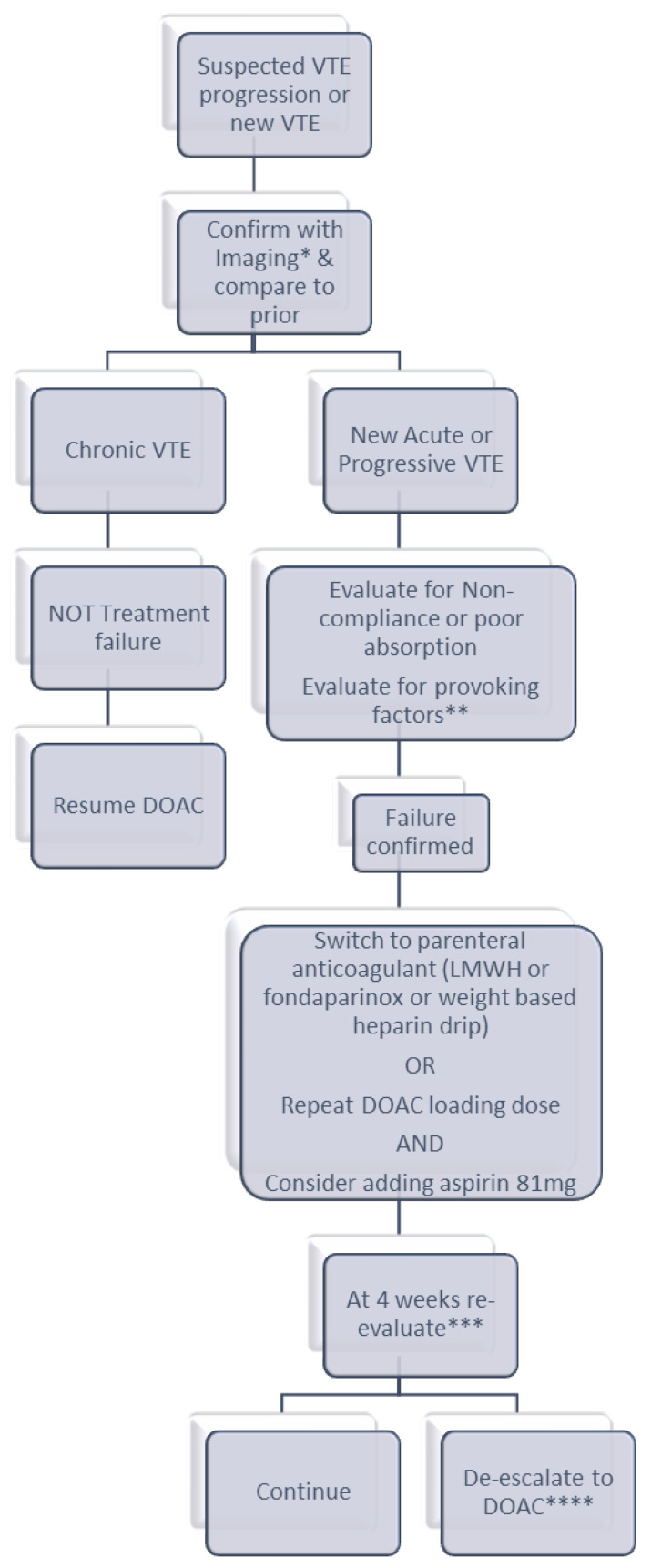
Proposed algorithm for suspected therapeutic-dose DOAC failure. * Workup to include the following: 4-limb Doppler US, CTA PE (based on symptoms or if not completed previously), CT A/P (extensive LLE proximal VTE), + ad hoc vascular imaging for unusual sites. Consider d-dimer in cases of non-diagnostic imaging. ** Transient provoking factors include surgery, hospitalization or immobility, indwelling lines/catheters, and COVID-19. Chronic provoking factors include malignancy, autoimmune disease, chronic infections, myeloproliferative disorders, and chronic infections. Consider hypercoagulable workup (FVL, PGM, protein C/S antigen and activity, antithrombin 3 and APLS labs, PNH and JAK2 if clinically relevant). *** Re-evaluation to include clinical evaluation and weighing risks/benefits; consider repeat imaging and assess the tolerance of current anticoagulant as well as bleeding risks for shared decision making with the patient. Patient preference for parenteral vs. oral anticoagulation in the long term considered here as well. **** Change to a different oral anticoagulant, choosing alternate DOAC or VKA, if the patient’s preference is for oral anticoagulation.

Lack of randomized controlled studies in this population is a challenge and makes our management recommendations empiric and subject to change as new information becomes available. Our practice is at a high-volume, tertiary care academic center. We report our group approach to these patients (see Figure 1), recognizing that these are not formal guidelines and find our experience helpful where there are limited formal recommendations otherwise.

We initiate our approach by confirming the diagnosis of recurrence with imaging based on the symptoms and locations of prior VTE in comparison to prior imaging where it is possible to confirm. Once recurrence is documented, we evaluate potential provoking factors, medication interactions or other factors that may contribute to recurrence. This approach also follows AHA/ACC/ACCP/ACEP/CHEST/SCAI/SHM/SIR/SVM/SVN and ISTH guideline recommendations for the evaluation of recurrent DVT or PE [1,22].

If there is a provoking factor or an anatomical cause for the VTE, our practice is to treat that and continue the DOAC. Evaluation for occult malignancy should also be considered for patients with breakthrough thrombosis—our practice is to complete all age-appropriate cancer screening with consideration for cross-sectional imaging to evaluate. We also have a low threshold to consider additional imaging to evaluate for anatomical causes in patients with recurrent upper extremity thrombosis in the absence of provoking causes (such as central venous access) and repetitive motion, as well as in patients with left lower extremity DVT meeting demographics concerning for May–Thurner syndrome. If an anatomical cause is identified, we request consultation by interventional radiology and/or vascular surgery to correct the anomaly.

If the recurrent VTE occurred while the patient was on half of the therapeutic dose (prophylactic dose) of a DOAC, we increase it to full (therapeutic) dose if there is not a contraindication to do so.

If the recurrent VTE occurred while the patient was on a therapeutic dose of DOAC, we believe the most logical management strategies include changing to an alternate parenteral anticoagulant (IV heparin, SQ LMWH, or fondaparinux) or changing to a loading dose of the DOAC. As described above, there are no randomized data specifically in this setting, but we feel this strategy makes clinical sense based on initial studies of DOACs that showed reduced VTE with increased dose. We consider adding an antiplatelet medication to patients with treatment failure (if not already taking prior to recurrent VTE); in most cases this would be with aspirin 81 mg. If these strategies fail, we consider combining two anticoagulants with different mechanisms of action. We reserve transition to VKA for patients with confirmed high-risk antiphospholipid antibody syndrome. In patients who have previously had breakthrough VTE on VKA, if otherwise consistently therapeutic INR is shown, we consider higher-intensity VKA (with INR goal range 2.5–3.5 or 3–4). These suggested dose-escalation strategies increase the risk of bleeding and must carefully be weighed against potential benefits. We prefer apixaban in this setting, based on the recent COBRRA study that showed lower risk of clinically relevant bleeding compared to rivaroxaban, with similar efficacy [40].

We re-evaluate patients after 4 weeks of therapy. It is often difficult to continue parenteral anticoagulants long term due to patient preference. In the absence of complications, we either continue the same with re-evaluation 1–3 months later or switch to a therapeutic dose of a DOAC for the long term. In making this decision, we consider the severity of the recurrent VTE, bleeding risk and comorbidities.

We recognize that there are limited data for combining DOACs with antiplatelet agents, specifically in the setting of DOAC failure. As noted above, limited data for the combination did not show reduced risk of recurrent VTE; however, only data from patients on antiplatelet medication prior to initiation of DOAC are available. As there are no randomized data to guide management in the setting of DOAC failure, we carefully weigh increased bleeding risk prior to addition of an antiplatelet agent to anticoagulation.

## 11. Conclusions and Future Direction

We have multiple anticoagulation options for front-line treatment of VTE, with the preferred option being a DOAC unless the patient has a contraindication. Unfortunately, when patients have recurrent VTE despite therapeutic anticoagulation, there is limited evidence to guide subsequent anticoagulation. We report a review of the available evidence and our practice at a high-volume academic center with a combined experience of approximately 100 author/years in consulting and managing these patients. Our suggested approach is also in line with recently published recommendations and guidelines that comment on recurrent pulmonary embolism despite anticoagulation [1,10,22]. Further data and prospective studies are needed to know the best management in these patients. We hope to use this framework to generate a database of our current practice and possible future prospective clinical trials.

## Figures and Tables

**Table 1:** Venous doppler findings of acute versus chronic VTE.

	Vein	Compressibility	Echogenicity	Collaterals	Occlusive *
Acute thrombosis	Distended	Partial or no compressibility	Hypoechoic thrombus	Absent	Occlusive
Chronic thrombosis	Narrow, irregular	Incompressible	Hyperechoic thrombus	Present	Non-occlusive

* Not all acute thromboses are occlusive; however, an occlusive thrombus without collaterals is supportive of acute rather than chronic VTE.

## Data Availability

No new data were created or analyzed in this study. Data sharing is not applicable to this article.
